# Core-Shell Structure Design of Hollow Mesoporous Silica Nanospheres Based on Thermo-Sensitive PNIPAM and pH-Responsive Catechol-Fe^3+^ Complex

**DOI:** 10.3390/polym11111832

**Published:** 2019-11-07

**Authors:** Weili Peng, Zeping Zhang, Minzhi Rong, Mingqiu Zhang

**Affiliations:** Key Laboratory for Polymeric Composite and Functional Materials of Ministry of Education, GD HPPC Lab, School of Chemistry, Sun Yat-sen University, Guangzhou 510275, China; pengweil@mail2.sysu.edu.cn (W.P.); ceszmq@mail.sysu.edu.cn (M.Z.)

**Keywords:** hollow mesoporous silica microspheres, thermo-responsive, pH-responsive, catechol-Fe^3+^, controlled release

## Abstract

A kind of core-shell hybrid nanoparticle comprised of a hollow mesoporous silica nanoparticles (HMS) core and a copolymer shell bearing N-(3,4-dihydroxyphenethyl) methacrylamide (DMA) and N-isopropylacrylamide (NIPAM) as responsive moieties was prepared. Moreover, the factors that could impact the surface morphology and hierarchical porous structure were discussed. In the presence of Fe^3+^, catechol-Fe^3+^ complexes were formed to achieve pH-responsive polymer shell, combining with thermal-sensitiveness of poly(N-isopropylacrylamide). Doxorubicin (DOX) was applied as a model drug and the behaviors of its loading/release behaviors were investigated to prove the idea. The results exhibited a significant drug loading capacity of 8.6% and embed efficiency of 94.6% under 1 mg ml^–1^ DOX/PBS solution. In fact, the loading capacity of drug can be easily improved to as high as 28.0% by increasing the DOX concentration. The vitro cytotoxicity assay also indicated that the as-prepared nanoparticles have no significant cytotoxicity on RAW 264.7 cells. The in vitro experiment showed that the cumulative release of DOX was obviously dependent on the temperature and pH values. This pH/temperature-sensitive hollow mesoporous silica nanosphere is expected to have potential applications in controlled drug release.

## 1. Introduction

The construction of smart delivery systems for drugs, such as polymeric hydrogels [1], micelles [2], liposomes [3], various inorganic [4], and organic-inorganic hybrid nanoparticles [5], has been attracting great attentions in recent years. Among them, hollow mesoporous silica (HMS) nanoparticles have the advantages of good biocompatibility, large hollow space, highly ordered mesopores, high surface areas, and easily functionalized surface, showing potential applications as an ideal carrier for delivery systems. [6,7] However, unmodified HMS lacks of controlled drug release ability, satisfactory dispersity, and stability, which highlights the importance of surface modification. [8] Numerous studies have shown that drug release from these sophisticatedly engineered multifunctional nanocontainers with appropriate surface modification could be commonly controlled by the triggering of time [9], pH [10,11], redox [12], temperature [13], and magnetic field [14]. It is widely known that tumor tissues have both lower pH value [15,16] and higher temperature [17] as compared to normal tissues. Consequently, pH– and temperature-responsive polymer-modified HMS that could open the pores in either lower pH or higher temperature should promote the drug release in specific tumor sites. Furthermore, previous researches have also indicated that the appropriate physical characteristic (e.g., stiffness, size and shape) of nanoparticles was beneficial for efficient drug delivery. [18,19]

Poly(N-isopropylacrylamide) (PNIPAM), as one of the most widely investigated temperature-responsive polymers, exhibits a phase transition abruptly at a lower critical solution temperature (LCST) of about 32 °C. Below the LCST, the polymer is in the coil (soluble) state with strong hydrogen bonding between the amide groups of PNIPAM and surrounding water molecules. Above the LCST, PNIPAM turns into a collapsed (insoluble) state due to the increase of hydrophobic association. This character has been widely utilized for thermo-responsive drug delivery [20,21].

Marine mussels are a fascinating species that can anchor themselves to the turbulent intertidal zone by secreting byssal adhesion protein to form an adhesive plaque. This class of proteins contains lots of 3,4-dihydroxyphenylalanine (DOPA) residues, which can form robust complexes with iron (III) ions (Fe^3+^). The catechol-Fe^3+^ coordination numbers have been widely proven to be adjustable in the order of mono-, bis-, and tris-catechol complex when the pH is increased from about 4 to 9. [22,23] Therefore, pH-responsive biomimetic drug delivery hydrogel can be designed on the basis of the above properties of catechol-Fe^3+^ coordination system. [1,24,25] However, there are few applications of this coordination chemistry in HMS surface modification and controlled drug release so far.

**Figure 1:**
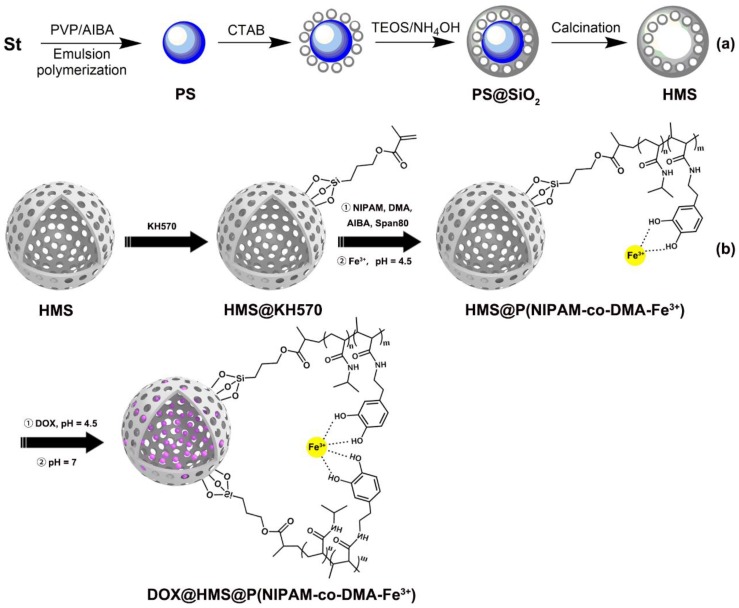

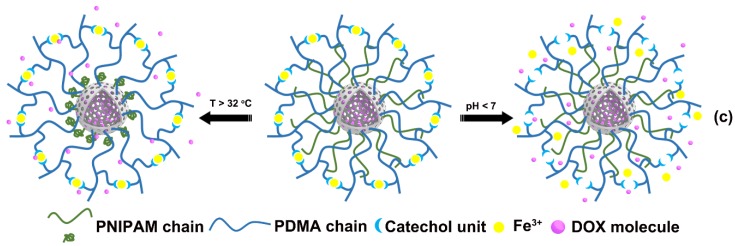
Schematic illustration of the synthesis process of (**a**) hollow mesoporous silica (HMS) and (**b**) doxorubicin (DOX)@HMS@P(NIPAM-co-DMA-Fe^3+^). (**c**) Schematic diagram of drug release.

Herein, HMS with abundant mesoporous structure were firstly prepared by template method (Figure 1a). Subsequently, the vinyl groups were incorporated into the surface of HMS by reacting with silane coupling agent KH570, followed by surface seed precipitation copolymerization to achieve linear poly(N-isopropylacrylamide-co-N-(3,4-dihydroxyphenethyl) methacrylamide) copolymer-grafted HMS (HMS@P(NIPAM-co-DMA)) (Figure 1b). Afterwards, the polymer shell was functionalized in the presence of Fe^3+^ to form the catechol-Fe^3+^ complex, leading to a pH/temperature-sensitive nanoparticle (HMS@P(NIPAM-co-DMA-Fe^3+^)). The morphology, structure, and structure-property relationship of unmodified and modified HMS are studied and characterized in detail. In addition, doxorubicin (DOX) was used as a model drug to prove the drug loading ability and pH/temperature responsive release behavior of the modified HMS.

## 2. Materials and Methods 

### 2.1. Materials

Styrene (St), tetraethoxysilane (TEOS), ammonia, sodium tetraborate, sodium bicarbonate, sodium hydroxide, anhydrous magnesium sulfate (MgSO_4_), Span80, potassium dihydrogen phosphate, sodium hydrogen phosphate, sodium chloride, and potassium chloride were purchased from Guangzhou Chemical Reagent Factory, Guangzhou, China. 2,2’-Azobis(2-methylpropionamidine) dihydrochloride (AIBA, 97%), hexadecyl trimethyl ammonium bromide (CTAB, 99%), polyvinylpyrrolidone (PVP, k29-32), dopamine (98%), methacrylic anhydride (94%), N-isopropylacrylamide (NIPAM, 98%), and 3-methacryloxyporpyltrimethoxysilane (KH570, 97%) were supplied by Shanghai Aladdin Biochemical Technology Co., Ltd., Shanghai, China. Doxorubicin (DOX) was obtained from Dalian Meilun Biotech Co., Ltd., Dalian, China. Styrene was purified by washing with alkaline and water, drying with anhydrous MgSO_4_, and vacuum distillation before use. All of the other chemicals and solvents were used as received.

### 2.2. Synthesis of Polystyrene (PS) Sphere Template

The monodisperse PS microspheres were synthesized by soap-free emulsion polymerization [26], as follows: 10.0 g St, 90.0 g H_2_O, and a certain amount of PVP (i.e., 6 g, 4.5 g, or 3 g) were added into a 250 mL three-necked flask that was equipped with a mechanical stirrer. Subsequently, the mixture was synchronously stirred and bubbled with argon for 30 min., followed by slowly heating to 60 °C. Subsequently, 10 ml aqueous solution of AIBA (0.26 g) was dropped into the above mixture with a constant pressure dropping funnel. Finally, the reaction was refluxed at 70 °C for 24 h under argon atmosphere. After completion of the polymerization, the resultant PS emulsions (solid content = 10 wt.%) with a different average diameter and the yield of about 80% were collected.

### 2.3. Synthesis of Hollow Mesoporous Silica Microspheres (HMS)

Silica-coated PS core-shell microsphere (PS@SiO_2_) and hollow mesoporous silica microspheres (HMS) were fabricated by referring to literatures [27,28] (Figure 1a). PS emulsion (10 g, 10 wt.%), CTAB (0.5 g), absolute ethanol (25 ml), and 10 mL of water were added into a 100 ml three-necked flask and ultrasonic dispersed for 15 min. Afterwards, the mixture was kept at 70 °C and stirred, followed by the addition of 5 ml TEOS/ethanol (0.2 g ml^–1^) solution within 30 min. (the mass ratio of TEOS to PS is 1). After reaction for 10 min., 3 ml of ammonia solution (0.1 mol L^–1^) was quickly added and stirred for another 8 h. Afterward, the silica-coated PS core-shell microsphere (PS@SiO_2_) were collected by repeatedly centrifugation and washing with 50 wt.% ethanol several times, followed by vacuum drying at 60 °C overnight. Hollow mesoporous silica microspheres (HMS) were obtained after calcination in air at 300 °C for 2 h and then at 550 °C for 5 h. Fourier transform infrared (FTIR, Thermo Nicolet Corporation, Madison, WI, USA, KBr, Figure S1): ν = 3340, 1630, 1090, 960, 800 cm^–1^. 

### 2.4. Synthesis of N-(3,4-dihydroxyphenethyl) Methacrylamide (DMA)

Typically, DMA was synthesized, as follows, by referring to the modified method [29] (Scheme S1). Sodium tetraborate (2.0 g), sodium bicarbonate (0.8 g), and 50 ml of distilled water were added to a 100 ml three-necked flask and stirred for 30 min. in a nitrogen atmosphere to remove oxygen. Subsequently, dopamine hydrochloride (1.0 g) and tetrahydrofuran (THF) solution of methacrylic anhydride (5 ml, 10 wt.%) were rapidly added to the reaction mixture, respectively. Afterwards, NaOH solution (1 mol L^–1^) was applied to adjust the pH to > 8 and kept reacting for 16 h. After reaction, the reaction mixture was extracted with ethyl acetate. The pH value of the collected water phase was adjusted to < 2 and further extracted with ethyl acetate for several times. The organic phase was washed with distilled water, followed by drying overnight with anhydrous MgSO_4_, filtering, and concentrating. A certain amount of n-hexane was added to the concentrate solution and kept stirring for 8 h at 0 °C. Whereafter, sage green powder with the yield of 55% was obtained by cold filtration and vacuum drying at 50 °C for 24 h. FTIR (KBr, Thermo Nicolet Corporation, Madison, WI, USA, Figure S3): ν = 3370, 3200, 3030, 2930, 2860, 1650, 1591, 1556, 1527, 1449 cm^–1^; Proton nuclear magnetic resonance (^1^H NMR, Bruker, Karlsruhe, Germany, 400 MHz, DMSO-d6, δ, Figure S4): 8.67 and 8.79 (s, 2H; OH), 7.97 (t, J = 5.2 Hz, H; NH), 6.58~6.44 (m, 3H; Ar-H), 5.31 and 5.63 (s, 2H; =CH_2_), 3.24 (q, J = 6.0 Hz, 2H; CH_2_), 2.56 (t, J = 7.6 Hz, 2H; CH_2_), 1.85 (s, 3H; CH_3_); Carbon 13 nuclear magnetic resonance (^13^C NMR, Bruker, Karlsruhe, Germany, 400 MHz, DMSO-d6, δ, Figure S5): 167.7, 145.5, 130.7, 119.6, 118.2, 116.4, 115.9, 41.4, 35.1, 19.1.

### 2.5. Preparation of the KH570-Modified Silica Particle

Piranha solution was firstly used to activate the hydroxyl groups since most of the hydroxyl groups on the HMS surface were inactivated after calcination [30]. The typical process was as follows: HMS was added to the mixture solution of concentrated sulfuric acid and hydrogen peroxide (volume ratio = 3:1), followed by ultrasonic dispersion and then refluxing at 100 °C for 2 h. Afterwards, the cool reaction mixture was centrifuged and then washed with ethanol and deionized water several times.

KH570 (0.2 ml) was firstly added to a toluene suspension (30.0 mL) of HMS (0.1 g). The reaction mixture was refluxed at 110 °C for 6 h under a nitrogen atmosphere. KH570-modified HMS was further purified by Soxhlet extraction with ethanol at 95 °C for 24 h after four cycles of centrifugation (12000 rpm) and ultrasonic washing with ethanol and water. Finally, HMS@KH570 was obtained after vacuum drying at 50 °C for 12 h (grafting yields = 14.8%). FTIR (KBr, Thermo Nicolet Corporation, Madison, USA, Figure S6): ν = 3370, 3200, 3030, 2930, 2860, 1650, 1591, 1556, 1527, 1449 cm^–1^.

### 2.6. Synthesis of HMS Nanoparticles Coated with Sensitive Polymer Shell

Monodisperse HMS@P(NIPAM-co-DMA-Fe^3+^) cross-linked nanoparticles were synthesized, as follows. Briefly, HMS@KH570 nanoparticles (0.1 g), NIPAM (0.8 g), DMA (0.2 g), and 50 ml alcohol/water (volume ratio = 1:4) solution were added into a 100 mL three-necked flask equipped with a reflux condensing tube. Subsequently, the reaction mixture was heated to 70 °C and bubbled with nitrogen for 0.5 h, followed by reacting for another 10 h in the presence of AIBA (0.04 g) as initiator. Afterwards, the resultant HMS@P(NIPAM-co-DMA) core-shell nanoparticles (grafting yields = 14.6%) were purified by centrifugation and then washed with absolute ethanol. 

Moreover, the unpurified mixture of HMS@P(NIPAM-co-DMA) was added into the 50 ml of FeCl_3_ containing PBS solution (n(Fe^3+^):n(DMA) = 1:2, pH = 4.5) for catechol-Fe^3+^ mono or bis-coordination in order to form catechol-Fe^3+^ coordination bonds. The mixture was stirred for 48 h at room temperature, followed by centrifugation and vacuum drying at 50 °C for 12 h to achieve HMS@P(NIPAM-co-DMA-Fe^3+^).

### 2.7. Characterization

UV-vis spectra were obtained while using a Perkin-Elmer Lambda 750 UV/vis spectrophotometer. Fourier transform infrared (FTIR, Thermo Nicolet Corporation, Madison, WI USA) analysis was conducted on a Nicolet Avatar 330 FT-IR spectroscopy. ^1^H NMR and ^13^C NMR spectra were measured by an AVANCE III 400MHz (400 MHz, Bruker, Karlsruhe, Germany) with DMSO-d6 as solvent. X-ray photoelectron spectroscopy (XPS, Thermo Fisher Scientific, Waltham, USA) spectra were measured on a Thermo SCIENTIFIC ESCALAB 250Xi. The products were visualized while using a JEM-2010HR transmission electron microscope (TEM, Japan Electronics Co., Ltd, Tokyo, Japan) and Hitachi S4800 scanning electron microscopy (SEM, Hitachi, Tokyo, Japan). Dynamic light scattering (DLS, Brookhaven, NY, USA) was conducted on a BI-200SM. The nitrogen adsorption/desorption isotherms and pore-structure analysis were conducted by an ASAP2020 surface area analyzer (Micromeritics Instrument Corporation, AT, USA) at 77 K. X-ray diffraction (XRD, Rigaku Corporation, Tokyo, Japan) patterns were obtained on a RIGAKU D-MAX 2200 VPC with Cu-Kα (λ = 1.54 Å) radiation. The particle zeta potentials were measured with a ZetaPALS zeta potential analyzer (Brookhaven, New York, USA). 

Temperature gravimetric Analysis (TGA, NETZSCH, Hanau, Germany) was conducted on a Netzsch TG-209 in N_2_ atmosphere. Grafting yields of KH570 and copolymer P(NIPAM-co-DMA) were calculated according to the following equations [31,32]:

GY = [(*m_a_*−*m_b_*)/*m_r_*] × 100% (1)

where *m_b_* and *m_a_* are the weight loss of nanoparticles between 250~800 °C before and after modification, respectively; *m_r_* is the residue weight of nanoparticles.

The cytotoxicity of HMS@P(NIPAM-co-DMA-Fe^3+^) was carried out on RAW 264.7 cells by MTT assay. Typically, RAW 264.7 cells that were cultured in RPMI 1640 medium with 10% FBS and 1% penicillin-streptomycin were seeded in 96-well plates at a density of 5000 cells/well. After incubating for 24 h in CO_2_ (5%) at 37 °C, fresh medium containing various amounts of HMS@P(NIPAM-co-DMA-Fe^3+^) was added. When the incubating time is 24 or 48 h, the cells were washed with PBS and then incubated with fresh medium containing MTT (0.5 mg ml^–1^) for 4 h. Afterwards, 200 μl of dimethyl sulfoxide (DMSO) was added to each well and the optical density was measured at 550 nm with a Multiskan FC microplate reader (Thermo Fisher Scientific, Waltham, WI, USA). The cell viability was calculated, as follows: cell viability = (OD_sample_/OD_control_)×100%, where the sample represents the cells that were treated with HMS@P(NIPAM-co-DMA-Fe^3+^) and the control means non-treated cells.

### 2.8. DOX loading capacity and controlled release behaviors in Vito

HMS@P(NIPAM-co-DMA-Fe^3+^) (50 mg) and 0.5 ml DOX/DMSO solution (10 mg ml^–1^) was added to 4.5 mL of PBS solution (pH = 4.5) and kept stirred at room temperature for 48 h. At a pH value of 4.5, the catechol-Fe^3+^ coordination number is 1, and thus the crosslinking density of copolymer is relatively lower, which is beneficial for the diffusion of DOX molecules into the nanoparticles. Subsequently, the pH value was adjusted to 7 to carry out crosslinking reaction, and thus kept DOX inside the nanoparticles. After three cycles of dispersion-centrifugation in water (pH = 7) and vacuum drying, DOX-loading nanoparticles (DOX@HMS@P(NIPAM-co-DMA-Fe^3+^)) were achieved.

The amount of free DOX in the collected supernatant was measured by the wavelength quantitative method using a UV spectrometer (Lambda 750 UV/vis) at 480 nm. The entrapment efficiency (EE) and loading capacity (LC) were calculated while using the following equations:

EE = [(*m_0_*−*m’*/*m_0_*] × 100% (2)

LC = [(*m_0_*−*m’*/*m_1_*] × 100% (3)

where *m_0_* and *m’* are the total mass of DOX and the mass of free DOX, respectively; *m_1_* is the mass of nanoparticle. 

Controlled release behavior was analyzed, as follows: DOX@HMS@P(NIPAM-co-DMA-Fe^3+^) (10 mg) was firstly dispersed in 1 mL of PBS and then transferred into a dialysis bag (molecular weight cut off = 14000). Afterwards, the dialysis bag was kept in 49 mL of PBS with various temperatures (26 °C and 37 °C) and pH (4.5, 6.0, and 7.4), which was placed in a water-bathing constant temperature vibrator (150 rev min.^–1^). At timed intervals, 5 mL of PBS buffer solution was sampled and analyzed by UV-vis. To keep a constant volume, 5 mL of fresh buffer was added after each sampling. All of the drug release results were averaged with three measurements.

## 3. Results

### 3.1. Optimizing the synthesis of HMS

**Figure 2:**
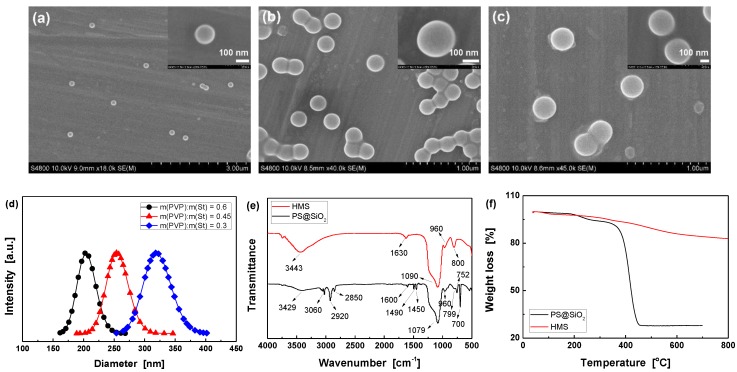
Scanning electron microscopy (SEM) images of polystyrene (PS) microspheres prepared at different mass ratio of polyvinylpyrrolidone (PVP) to St, (**a**) 0.6, (**b**) 0.45, (**c**) 0.3; The insets are enlarged images; The scale bars are 100 nm; (**d**) Hydrodynamic diameters of PS microspheres; (**e**) FT-IR spectra; and, (**f**) Temperature Gravimetric Analysis (TGA) curves of PS@SiO_2_ and HMS (preparation conditions: m(CTAB):m(TEOS):m(PS) = 0.5:1:1).

PS microspheres with various average diameter were firstly obtained by varying the mass ratio of PVP dispersant to St monomer. The particle size increase with decreasing mass ratio from 0.6 to 0.3, which are 145, 212, and 285 nm, as demonstrated by SEM (Figure 2a–c). Moreover, hydrodynamic diameters characterized by DLS are slightly larger than those of SEM [26,33]. Figure 2d shows that they are 203.9±3.5, 254.3±4.1, and 319.3±4.2 nm, respectively. The above results imply that the resultant PS microspheres have uniform particle size, smooth surface, and good mono-dispersity (polydispersity index of three kinds of PS microspheres are 0.005±0.0008, 0.006±0.0007, and 0.005±0.001, respectively) [34,35].

**Figure 3:**
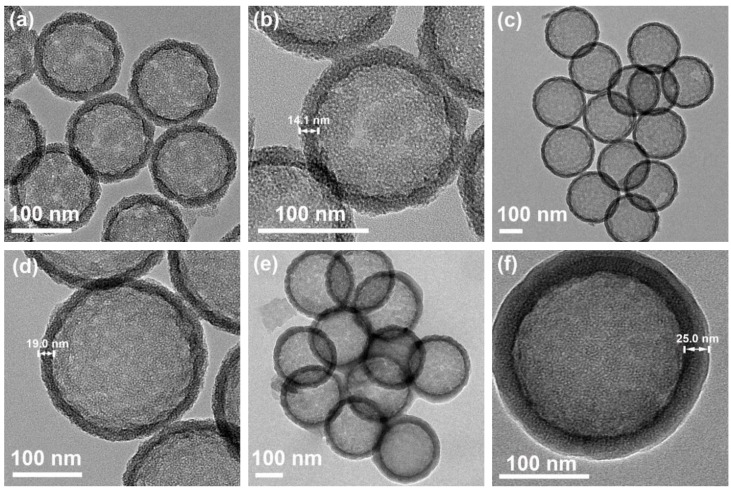
Transmission electron microscope (TEM) images of HMS obtained from 145 nm (**a**,**b**) and 212 nm (**c**,**d**) PS template microsphere, respectively; Preparation conditions: m(CTAB):m(TEOS):m(PS) = 0.5:1:1; (**e**,**f**) TEM image of HMS@P(NIPAM-co-DMA) obtained from (**c**,**d**).

As compared to PS@SiO_2_, the FTIR spectrum of HMS shows no characteristic peaks of organic compounds (Figure 2e), and there is only a small amount of weight loss in its TGA curve due to the dehydration of active hydroxyl groups on the surface of HMS (Figure 2f). The FTIR (Figure 2e) and TGA (Figure 2f) results both proved that the PS core and CTAB micelles in the shell were successfully removed after calcination [28,34]. Moreover, the XRD pattern of HMS in Figure S1 only exhibits a board diffraction peak centered at 2θ = 2.46°, corresponding to a repeating unit (i.e., pore spacing) of 3.59 nm [26]. It suggests the existence of mesopores with lower degree of order in the walls of the spheres, which is in agreement with the reported results [21,26,36]. Meanwhile, HMS also display a wide dispersion peak at 2θ = 20~30° that belongs to amorphous SiO_2_ nanoparticles [28,36]. SEM and TEM observations (Figure 3) indicate the excellent particle uniformity and the typical silica shell comprised of the mesoporous structure. Furthermore, the electron contrast between the cores and the shells in the high-resolution TEM images confirms the formation of hollow particles.

Moreover, monodispersed HMS with a tunable particle size and shell thickness were successfully achieved by varying diameter of PS microsphere and TEOS content. With 145 nm and 212 nm PS microspheres as templates, it is found that the diameters of resultant HMS are 157 nm and 245 nm (Figure S2a,b) when the mass ratio of PS to TEOS is 1 (i.e., m(TEOS):m(PS) = 1). High-resolution TEM images (Figure 3a–d) further identify that the diameters are 152 nm and 228 nm and the shell thicknesses are 14.1 nm and 19.0 nm, respectively. When TEOS content was increased to 1.5 times of PS (i.e., m(TEOS):m(PS) = 1.5), the shell thickness of HMS while using PS template with a diameter of 212 nm is more than 40 nm, which is estimated from the diameter obtained by SEM (Figure S2c). It is consistent with the previous studies of HMS based on PS-co-PVP microsphere [28].

**Figure 4:**
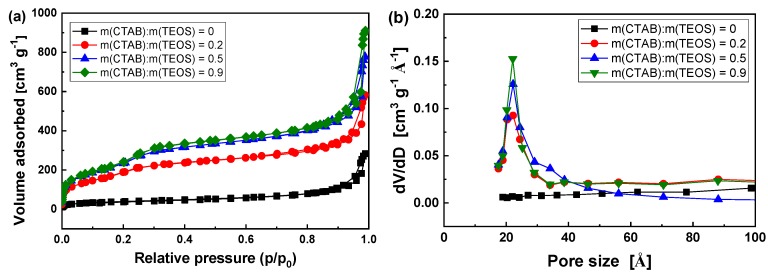
Nitrogen adsorption-desorption isotherms (**a**) and the pore size (**b**) of HMS prepared with various ratio of hexadecyl trimethyl ammonium bromide (CTAB) and tetraethoxysilane (TEOS).

Meanwhile, the content of CTAB plays an important role in controlling the morphologies, specific surface area (*S_BET_*), and total pore volume (*V_tot_*) of HMS. Figure 4 reveals a type IV physisorption isotherm with an increase in nitrogen uptake at a high relative pressure (p/p_0_) 0.9–1.0 and a hysteresis loop at p/p_0_ 0.8–1.0, indicating that HMS possesses a mesoporous structure [37,38]. The corresponding pore size distribution curves show a narrow pore distribution with a mean value of 2.2~2.3 nm. When the mass ratios of CTAB to TEOS is 0.5, the specific surface area and the average pore diameter are estimated to be about 866 m^2^ g^–1^ and 2.3 nm while using the BET and BJH methods, respectively. The specific surface area is comparable to, or even superior to, the similar product, as reported in these literatures [28,39]. In the mass ratio ranging from 0 to 0.9, the increase of CTAB could improve the specific surface area (144~895 m^3^ g^–1^) and total pore volume (0.072~1.482 cm^3^ g^-1^), but it has little effect on the pore diameter (Table S1). The regulatory functions of CTAB shown above are consistent with previous studies [40].

### 3.2. Preparation of HMS@P(NIPAM-co-DMA)

**Figure 5:**
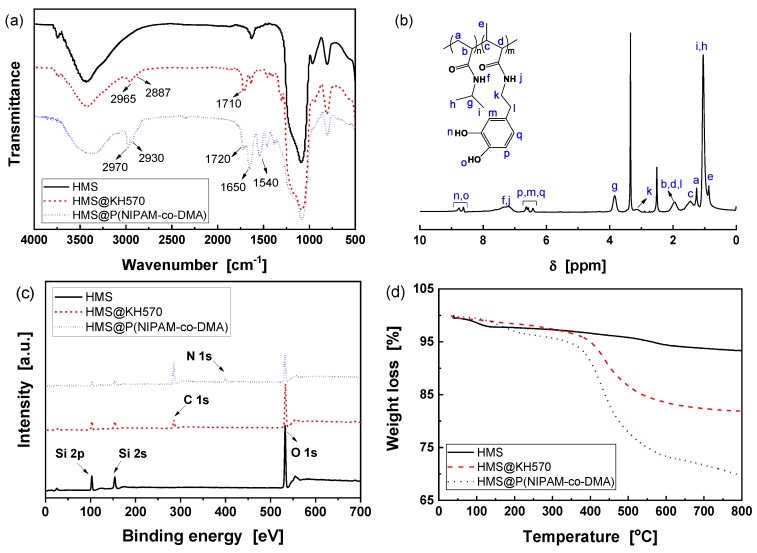

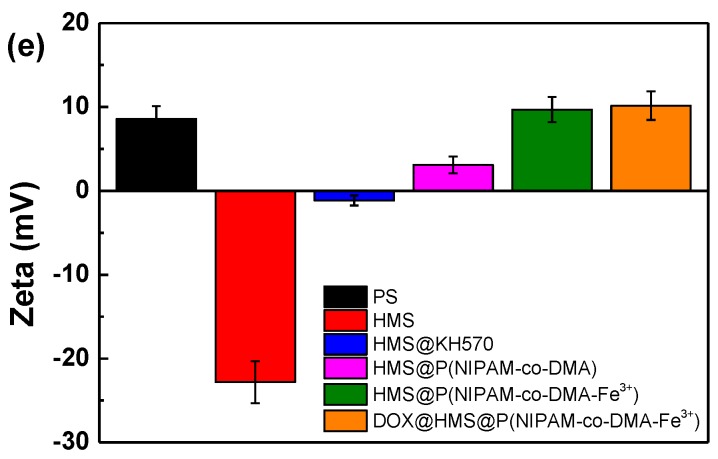
(**a**) FT-IR, (**b**) ^1^H NMR, (**c**) X-ray photoelectron spectroscopy (XPS), (**d**) TGA spectra and (**e**) zeta potentials of HMS, HMS@KH570 and HMS@P(NIPAM-co-DMA).

Before incorporating temperature and pH responsive functional copolymer into HMS nanospheres, HMS with a diameter of 245 nm (Figure 3c,d) was modified by silane coupling agent KH570 to introduce reactive double bonds. The obvious vibration absorptions of methyl and methylene in the range of 2800~3000 cm^–1^ and carbonyl at 1710 cm^–1^ indicate that KH570 has been successfully grafted onto HMS surface (Figure 5a) [41]. Subsequently, KH570 modified HMS was used as the seed and copolymerized with NIPAM and DMA. Accordingly, in the resulting product, the C=O stretching (1650 cm^–1^) and N-H bending (1540 cm^–1^) vibration absorptions of amide group in NIPAM [42], as well as C=O stretching (1720 cm^–1^) vibration absorption of ester group in DMA, can be observed, which implies the successful grafting of Poly(NIPAM-co-DMA) on the surface of HMS (Figure 5a). Furthermore, ^1^H NMR detected the chemical structure of copolymer after the removal of HMS component by treating with 20 vol.% hydrofluoric acid solution. As shown in Figure 5b, it shows all of the proton chemical shifts of the copolymers.

XPS analysis can provide insights into the chemical compositions of polymer shell [43]. Figure 5c shows the chemical identification of the surfaces of HMS, HMS@KH570 and HMS@P(NIPAM-co-DMA). The full spectrum reveals the presence of Si 2s, Si 2p, and O 1s emissions with binding energies at 154, 105, and 533 eV on the surface of HMS. After modification with KH570, the C 1s hydrocarbon peak at 285 eV is observed. With respect to HMS@P(NIPAM-co-DMA), the relative intensity of C 1s peak obviously increases, and a new peak at 399 eV corresponding to N 1s generated from the copolymer shell is obtained, which implies that NIPAM and DMA monomers were chemically grafted on HMS surface.

Subsequently, the zeta potentials of the as-prepared nanoparticles and DOX loaded HSM were measured. The results (Figure 5e) indicate that HMS possesses a negative zeta potential due to the strong electronegativity of Si–OH groups on the surface of nanoparticles. After grafting of KH-570, the Si–OH groups were reacted, leading to a low negative zeta potential. With the further incorporating of P(NIPAM-co-DMA) copolymer and Fe^3+^ ions, the zeta potential gradually becomes positive. It may be attributed to the fact that the copolymer and Fe^3+^ ions possess positive charge.

The weight loss of different nanoparticles in the range of 50~800 °C were measured by TGA in order to quantitatively determine the amount of grafted polymer (Figure 5d), and Table 1 summarizes the results. All of the samples present two stages of weight loss. For HMS, the weight loss stage below 250 °C is attributed to the evaporation of physically adsorbed water and residual solvent, while the decomposition of the silica-bonded hydroxyl and alkyl groups must be responsible for that between 250~800 °C [28,44]. After the grafting of copolymer, the latter weight loss stage of HMS@KH570 and HMS@P(NIPAM-co-DMA) increase from 4.1% of HMS to 16.2% and 26.4%, respectively. According to Equation (1), the grafting yields of KH570 and P(NIPAM-co-DMA) copolymer are estimated to be 14.8% and 14.6%, respectively. The free radical polymerization that was utilized in this work is a common method for introducing polymer on silica surface, but the grafting efficiency is relatively lower. As compared with grafting polymers that were obtained from ATRP and RAFT [45,46], the dispersity of the as-prepared P(NIPAM-co-DMA) random copolymer is higher, but the preparation condition is simpler.

The morphology and structure of the as-synthesized copolymer-grafted HMS was examined by means of TEM (Figure 3e,f), which confirmed the particle uniformity and typical core-shell structure. The results show that the diameter and shell thickness increase from 228 and 19.0 nm (Figure 3d) of HMS to 250 and 25.0 nm, respectively.

### 3.3. DOX Loading and Controlled Release in Vitro

**Figure 6:**
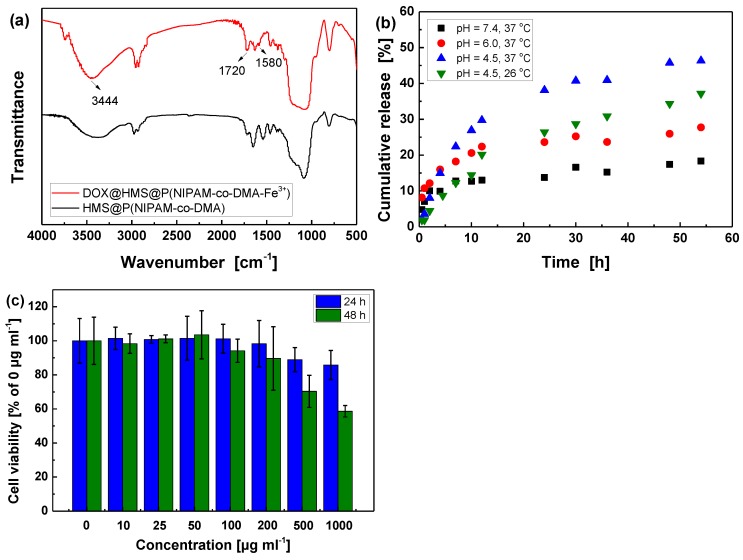
(**a**) FT-IR spectra of HMS@P(NIPAM-co-DMA) and DOX@HMS@P(NIPAM-co-DMA-Fe^3+^); (**b**) Cumulative release of DOX from DOX@HMS@P(NIPAM-co-DMA-Fe^3+^) nanoparticles in different conditions; (**c**) Cell viability of RAW 264.7 cells treated with different concentrations of HMS@P(NIPAM-co-DMA-Fe^3+^) for 24 h and 48 h.

Generally, the size of drug molecules should be smaller than the diameter of mesopore channels to achieve the effective storage and release of drugs in hollow mesoporous silica nanoparticles [47]. As one of the most potent anticancer drugs [48], DOX was applied as a model drug to evaluate the loading and controlled release properties of the modified HMS. The loading process of DOX is mainly based on the physical adsorption mechanism in mesoporous channels. In general, the incorporation of DOX was achieved by soaking HMS@P(NIPAM-co-DMA-Fe^3+^) nanoparticles in a DOX/PBS solution at a pH value of 4.5 for 48 h, accompanied by magnetic stirring to favor the diffusion of drug molecules through the mesoporous shell into the cavity. The intensities of vibration peaks at about 3444 (–OH and –NH_2_) and 1720 cm^–1^ (C=O) correspondingly increase for the loaded particles since the DOX molecule contains a large number of hydroxyl (–OH), amino (–NH_2_) and carbonyl (C=O) groups [49] (Figure 6a). Moreover, a new peak at 1580 cm^−1^ belonging to stretching vibration of -NH_2_ is observed, which also implies that DOX was successfully loaded. Moreover, the zeta potential of DOX-loaded nanoparticle (about 10.2 mV) was almost the same as that of HMS@P(NIPAM-co-DMA-Fe^3+^) (9.7 mV). In the loading process of DOX, the pH value was adjusted to 7 by hydrochloric acid finally, so the DOX molecule was electric neutral and it had little effect on zeta potential. 

Subsequently, the entrapment efficiency (EE), loading capacity (LC), mass percentage of drug were analyzed according to UV-vis standard curve (Figure S6) and Equations (2) and (3). As shown in Table 2, they are calculated to be 94.6%, 9.5 wt%, and 8.6 wt% under 1 mg ml^–1^ DOX/PBS solution, respectively. Importantly, the results show that the grafting polymer shell did not significantly affect the loading capacity of porous microspheres in comparation with unmodified HMS. Moreover, the TGA curves (Figure S7) also validate that the weight percentage of the drug is about 8.0 wt%, which is in agreement with the results of UV-vis experiment. In fact, the mass percentage of drug can be easily improved to as high as 28.0% by increasing the DOX concentration (e.g., 5 mg ml^–1^, Figure S7). The loading capacity and entrapment efficiency of modified HMS can both be comparable to the reported results by Yang and coworkers [50].

As shown in Figure 6b, the DOX release experiments were performed in PBS solution with various conditions to evaluate the pH and thermo-sensitivity of biomimetic HMS@P(NIPAM-co-DMA-Fe^3+^) nanoparticles. In the drug release process, DOX must diffuse across the polymer shell limited by crosslinked density and phase state of grafted polymers. Consequently, it is noticed that the release of DOX at 37 °C is effectively accelerated as the pH of the PBS buffer decreases. The release of DOX at pH values of 4.5, 6.0 and 7.4 for 48 h reach 45.8%, 30.0%, and 17.4%, respectively. It has been widely verified that the mono-, bis-, and tris-coordinated catechol-Fe^3+^ complexes form in turn when the pH is gradually increased from about 4 to 9 [22,23]. Since the crosslinking degree between the grafted copolymerization chains is controlled by the pH-sensitive catechol-Fe^3+^ coordination bonds, it consistently decreases with the decreasing of pH value, resulting in faster diffusion and release of drug within the same period of time. 

Moreover, 37 °C and 26 °C, being slightly higher and lower than LCST (32 °C) of the PNIPAM, were chosen to verify the temperature response behavior. The cumulative release of DOX within 48 h at 37 °C and pH value of 4.5 (i.e., 45.8%) is higher than that at 26 °C (i.e., 37.2%). The DOX release rate increase with temperature is ascribed to the shrunken state of the PNIPAM polymer shell and small hydrodynamic diameter above LCST, which is conductive to exposing the pore channels of HMS and shortening the diffusion pathways for faster release [21]. Hence, if these nanocarriers accumulate at a focus of infection with lower pH and elevated temperature, a faster local drug release might be expected [50]. For comparison, the reference sample DOX@HMS without polymer shell exhibits obvious burst release characteristics, which release 43.4% of DOX within 0.5 h (Figure S8). Drug release studies in vitro show that the release behavior is pH and temperature-responsive, and comparable to other reported drug delivery system [49,51], displaying the potential application prospects in the field of drug sustained release. However, the biocompatibility and antitumorigenic effect need to be carefully studied in the future.

### 3.4. In Vitro Cytotoxicity Assay

The cytotoxicity of nanoparticles is a crucial issue for their controlled drug release applications. [49] For evaluating cytotoxicity of the as-prepared HMS@P(NIPAM-co-DMA-Fe^3+^), cell viability was measured by MTT assay. As shown in Figure 6c, when the incubation time is 24 h, the survival of the treated RAW 264.7 cells was more than 85% relative to the untreated cells, even at 1000 μg ml^–1^ of HMS@P(NIPAM-co-DMA-Fe^3+^). With the extending of incubation time to 48 h, the viability of cells treated with high concentration of HMS@P(NIPAM-co-DMA-Fe^3+^) (e.g., > 500 μg ml^–1^) decreases sharply, but the cell viability remains over 90% within 100 μg ml^–1^ of nanoparticles. The results indicate that HMS@P(NIPAM-co-DMA-Fe^3+^) nanoparticles have no significant cytotoxicity on RAW 264.7 cells, and they present good biocompatibility as drug carriers. It is in a good agreement with the previous results of the low cytotoxicity of nanospheres based on silica, PNIPAM, and polydopamine, respectively. [24,25,52,53]

## 4. Conclusions

In summary, a biomimetic composite microsphere with HMS core and P(NIPAM-co-DMA) shell was firstly prepared. Especially, with the addition of Fe^3+^, the polymer shell of HMS@P(NIPAM-co-DMA) can be crosslinked by catechol-Fe^3+^ coordination bonds. As a result, the polymer shell possesses both temperature and pH sensitivity, which is dependent on dissociation/association of the coordination bonds combining with phase transition behavior of PNIPAM. DOX was applied as a model drug for evaluating the loading and controlled release properties. The results demonstrated that this composite microsphere had a significant drug loading capacity of 8.84% and embed efficiency of 94.60% under 1 mg ml^–1^ DOX/PBS solution. The cumulative release of DOX in vitro (37 °C) showed low leakage (17.4%) at a pH value of 7.4, but it was significantly enhanced to 45.8% at the pH value of 4.5. Moreover, the cumulative release at 37 °C is faster than that at 26 °C. The vitro cytotoxicity assay also indicated that the as-prepared nanoparticles have no significant cytotoxicity on RAW 264.7 cells. These results demonstrated that the drug release was apparently environmentally responsive and has potential application in controlled drug release.

## Figures and Tables

**Table 1:** TGA results and grafting yield for various nanoparticles obtained from Figure 5d.

Sample	Weight Loss < 250 °C [%]	Weight Loss at 250~800 °C [%]	Residue Weight [%]	Grafting Yield [%]
HMS	2.5	4.1	93.4	-
HMS@KH570	2.0	16.2	81.8	14.8
HMS@P(NIPAM-co-DMA)	3.9	26.4	69.7	14.6

**Table 2:** Comparisons of drug loading properties under 1 mg ml^–1^ DOX/PBS solution calculated from Figures S6 and S7.

Sample	Drug loading ^1^ [mg]	Loading capacity ^1^ (LC) [%]	Mass percentage ^1^ [%]	Entrapment efficiency ^1^ (EE) [%]	Mass percentage ^2^ [%]
HMS	4.70	9.4	8.5	94.0	8.1
HMS@P(NIPAM-co-DMA-Fe^3+^)	4.73	9.5	8.6	94.6	8.0

^1^ obtained from UV-vis standard curves; ^2^ obtained from TGA curves.

## Supplementary Materials

The following are available online at https://www.mdpi.com/2073-4360/11/11/1832/s1, Figure S1: Powder XRD patterns of HMS, Figure S2: SEM images of HMS obtained from 145 nm (a) and 212 nm (b) PS template microsphere, Figure S3: FT-IR spectra of DMA, Figure S4: ^1^H NMR spectra of DMA in DMSO-d6, Figure S5: ^13^C NMR spectra of DMA in DMSO-d6, Figure S6: The standard curve of DOX obtained from ultraviolet absorption at 480 nm in the concentration range of 0~10 µg ml^-1^ (a) and 20~90 µg ml^-1^ (b), Figure S7: TGA curves of (a) DOX@HMS@P(NIPAM-co-DMA) and un-modified HMS (b) obtained from DOX/PBS with different concentrations, Figure S8: Cumulative release of DOX from DOX@HMS, Scheme S1: The synthesis mechanism of DMA, Table S1: Structure properties of HMS prepared with various ratio of CTAB and TEOS.
