# Supplementary material for: Core-Shell Structure Design of Hollow Mesoporous Silica Nanospheres Based on Thermo-Sensitive PNIPAM and pH-Responsive Catechol-Fe3+ Complex
Source: Polymers (Basel). 2019 Nov 7;11(11):1832. doi: 10.3390/polym11111832 (PMC6918372; doi:10.3390/polym11111832)
Supplement: Supplementary file 1 [file polymers-11-01832-s001.pdf]

Supporting information

Core-shell structure design of hollow mesoporous silica nanospheres for dual pH/thermo-sensitivity

Peng Wei Li, Zhang Ze Ping*, Rong Min Zhi* and Zhang Ming Qiu
Key Laboratory for Polymeric Composite and Functional Materials of Ministry of Education, GD HPPC Lab, School of Chemistry, Sun Yat-sen University, Guangzhou 510275, P. R. China
Email: 357386053@qq.com, cesrmz@mail.sysu.edu.cn

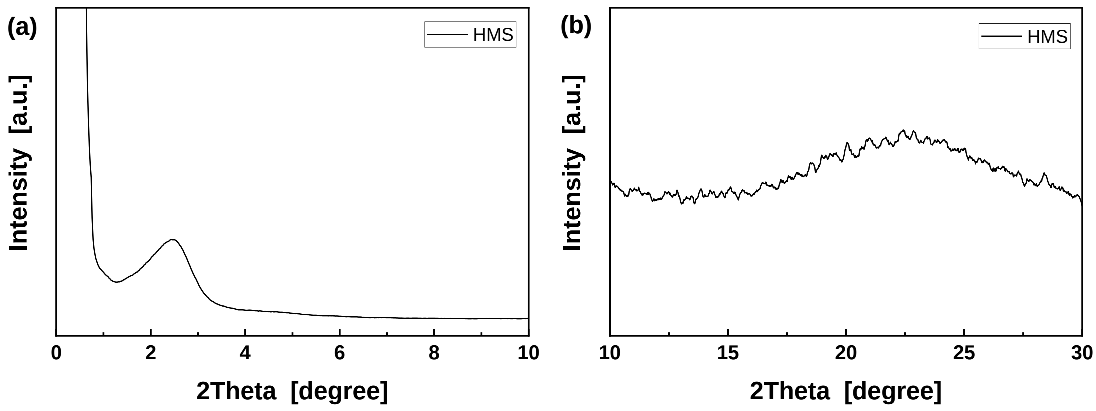

Figure S1. Powder XRD patterns of HMS (preparation conditions: m(CTAB):m(TEOS):m(PS) = 0.5:1:1).

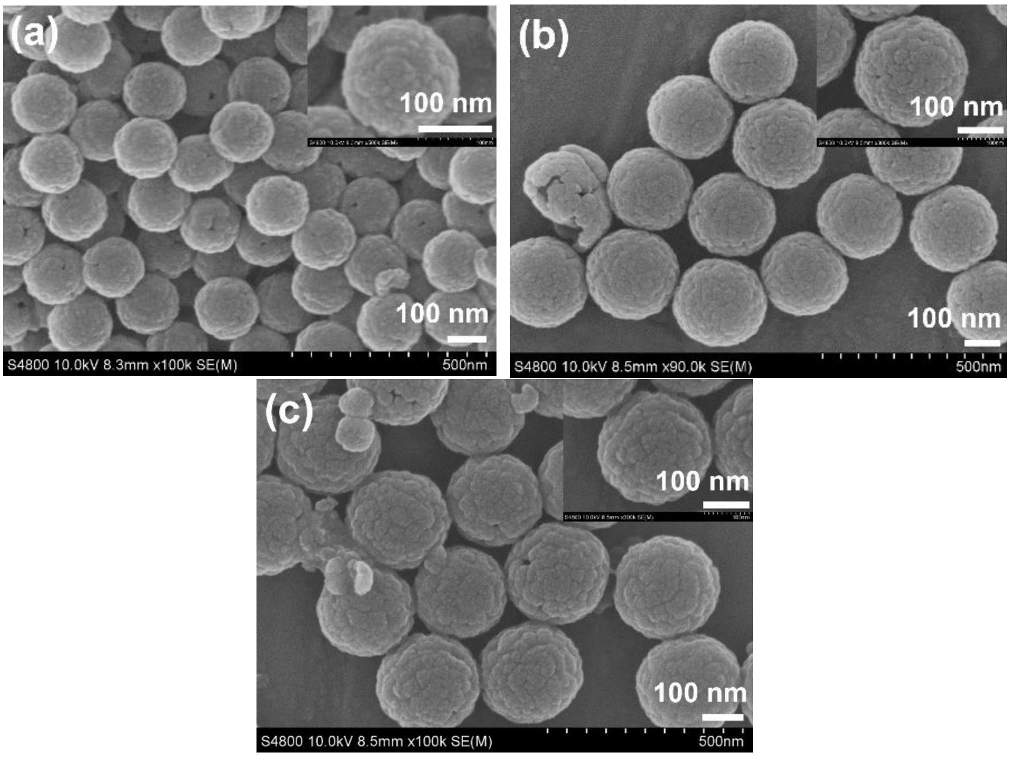

Figure S2. SEM images of HMS obtained from 145 nm (a) and 212 nm (b) PS template microsphere, respectively. Preparation conditions: m(CTAB):m(TEOS):m(PS) = 0.5:1:1. (c) SEM images of HMS obtained from 212 nm PS template microsphere. Preparation conditions: m(CTAB):m(TEOS):m(PS) = 0.5:1.5:1.

Table S1. Structure properties of HMS prepared with various ratio of CTAB and TEOS

m(CTAB):m(TEOS)	S_{BET} (m^2 g^{-1})	V_{tot} (cm^3 g^{-1})	Pore size (nm)
0	144	0.072	-
0.2	715	0.901	2.2

0.5	866	1.203	2.3
0.9	895	1.482	2.2

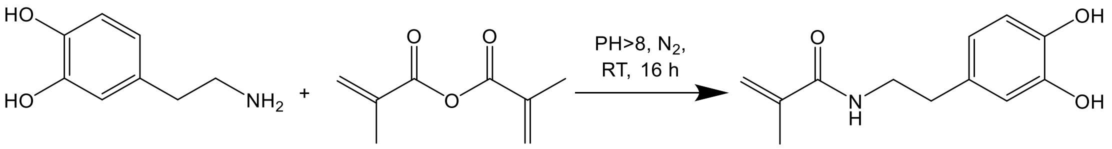

Scheme S1. The synthesis mechanism of DMA.

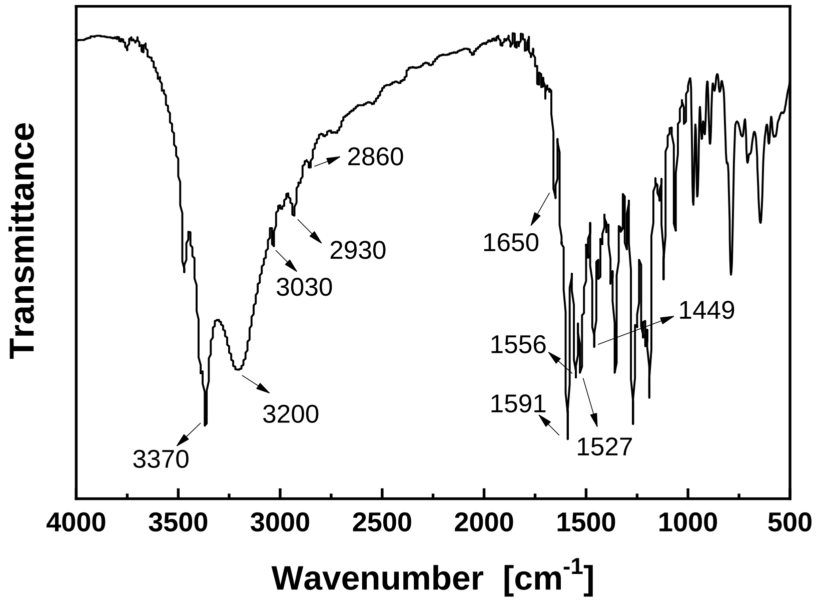

Figure S3. FT-IR spectra of DMA.

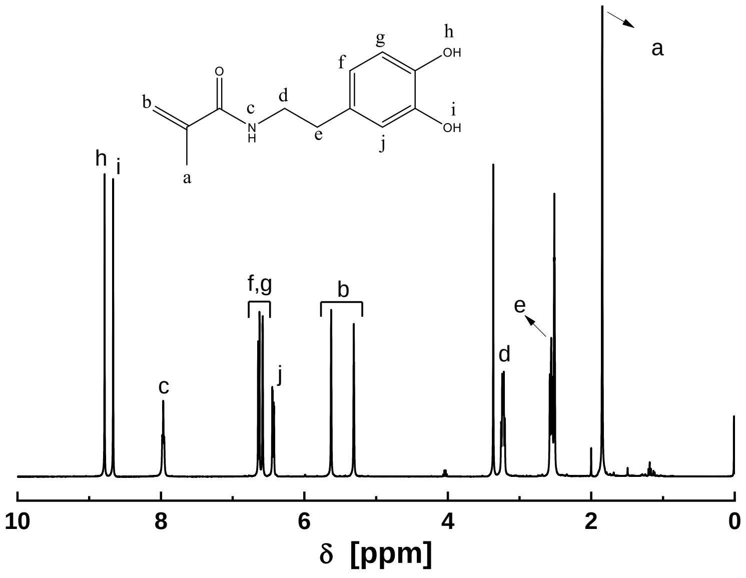

Figure S4. ^{1}H NMR spectra of DMA in DMSO-*d*6.

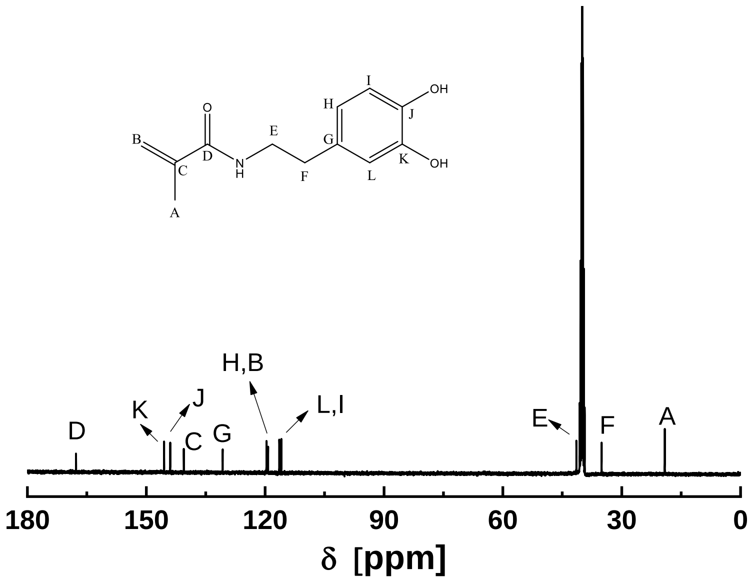

Figure S5. ^{13}C NMR spectra of DMA in DMSO-*d6*.

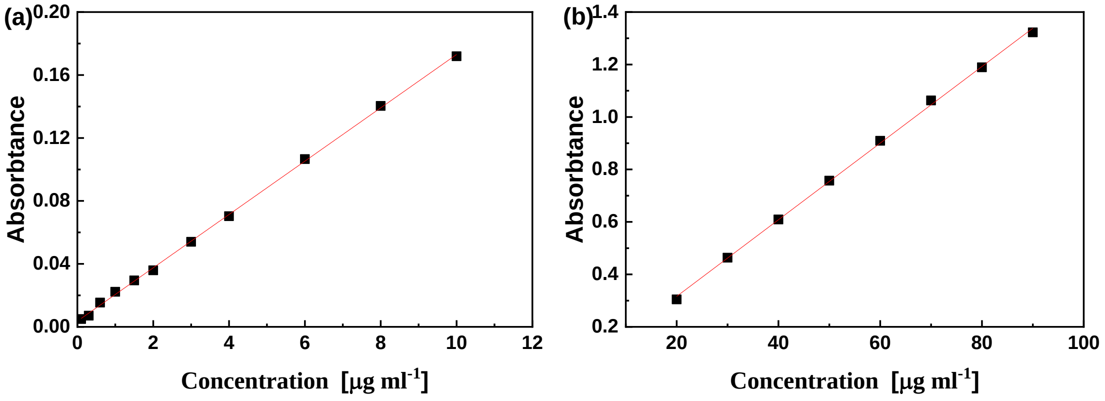

Figure S6. The standard curve of DOX obtained from ultraviolet absorption at 480 nm in the concentration range of 0~10 μg ml^{-1} (a) and 20~90 μg ml^{-1} (b).

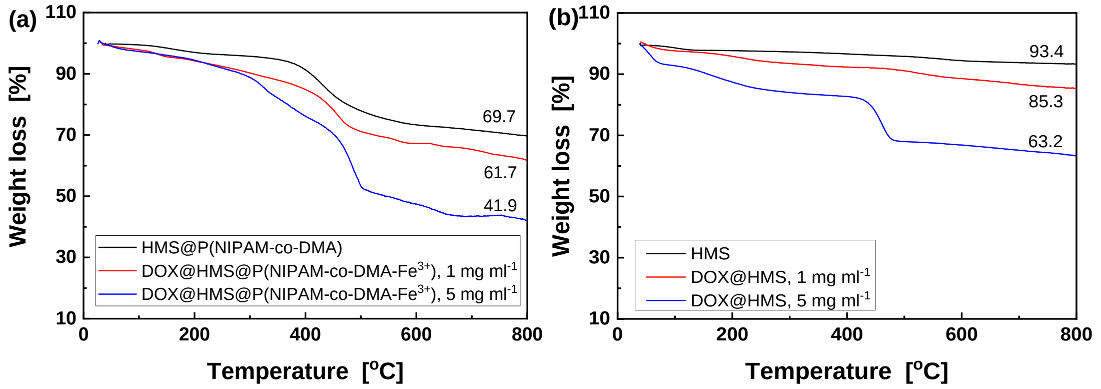

Figure S7. TGA curves of (a) DOX@HMS@P(NIPAM-co-DMA) and un-modified HMS (b) obtained from DOX/PBS of different concentrations.

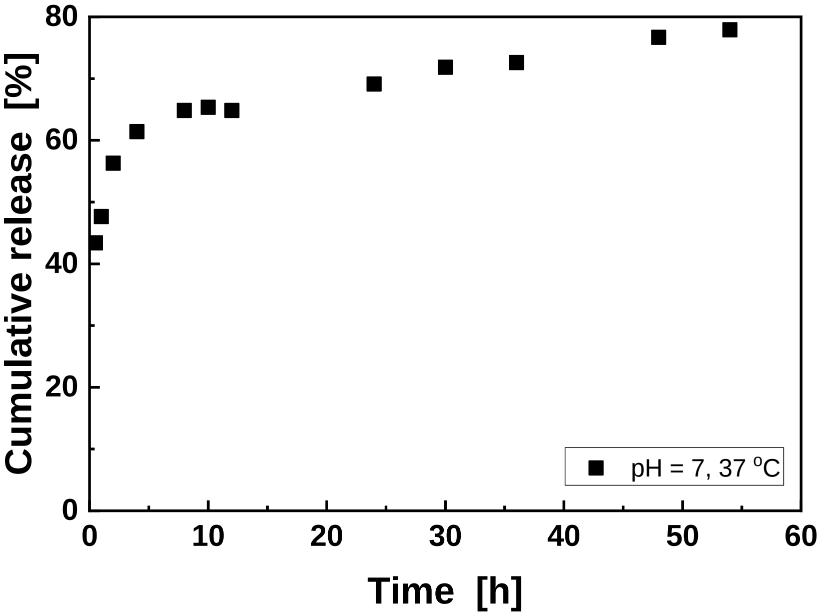

Figure S8. Cumulative release of DOX from DOX@HMS.
